# Development of a new biocathode for a single enzyme biofuel cell fuelled by glucose

**DOI:** 10.1038/s41598-021-97488-w

**Published:** 2021-09-17

**Authors:** Asta Kausaite-Minkstimiene, Algimantas Kaminskas, Anton Popov, Arunas Ramanavicius, Almira Ramanaviciene

**Affiliations:** 1 Nanotechnas – Center of Nanotechnology and Materials Science, Institute of Chemistry, Faculty of Chemistry and Geosciences, Vilnius University, Naugarduko st. 24, 03225 Vilnius, Lithuania; 2 Department of Physical Chemistry, Institute of Chemistry, Faculty of Chemistry and Geosciences, Vilnius University, Naugarduko st. 24, 03225 Vilnius, Lithuania

**Keywords:** Biocatalysis, Fuel cells

## Abstract

In this study, we reported the development of Prussian blue (PB), poly(pyrrole-2-carboxylic acid) (PPCA), and glucose oxidase (GOx) biocomposite modified graphite rod (GR) electrode as a potential biocathode for single enzyme biofuel cell fuelled by glucose. In order to design the biocathode, the GR electrode was coated with a composite of PB particles embedded in the PPCA shell and an additional layer of PPCA by cyclic voltammetry. Meanwhile, GOx molecules were covalently attached to the carboxyl groups of PPCA by an amide bond. The optimal conditions for the biocathode preparation were elaborated experimentally. After optimization, the developed biocathode showed excellent electrocatalytic activity toward the reduction of H_2_O_2_ formed during GOx catalyzed glucose oxidation at a low potential of 0.1 V vs Ag/AgCl, as well as good electrochemical performance. An electrocatalytic current density of 31.68 ± 2.70 μA/cm^2^ and open-circuit potential (OCP) of 293.34 ± 15.70 mV in O_2_-saturated 10 mM glucose solution at pH 6.0 were recorded. A maximal OCP of 430.15 ± 15.10 mV was recorded at 98.86 mM of glucose. In addition, the biocathode showed good operational stability, maintaining 95.53 ± 0.15% of the initial response after 14 days. These results suggest that this simply designed biocathode can be applied to the construction of a glucose-powered single enzyme biofuel cell.

## Introduction

With the growing demand for green electrical energy generation technologies, scientists are making great efforts to develop fuel cells (FCs), which are considered as one of the most promising alternative sustainable energy sources due to their renewable and environmental protection characteristics^1^. Unlike conventional FCs, which utilize the oxidation of fuels (H_2_, ethanol, or methanol) on an anode and reduction of an oxidant on a cathode employing a noble metal catalyst^2^, biological fuel cells (biofuel cells, BFCs) convert chemical energy into electrical energy by using organic fuels (sugars, alcohols, organic acids) produced during metabolic processes and a biological catalyst, which is usually either a microorganism or an enzyme. An enzymatic biofuel cells (EBFCs) are type of BFCs that use purified redox enzymes immobilized on an anode and/or on a cathode to achieve electrocatalytic reactions^3^. Enzymes are highly specific to their respective substrate and typically operates in mild conditions. Therefore, BFCs are an attractive alternative when it is not possible to use high temperatures or where harsh reaction conditions are undesirable. Moreover, enzymes immobilized on the electrode surface allow membrane-less configuration of EBFCs, opening up opportunities for the development of miniaturized systems for powering electronic devices^4^ and self-powered electrochemical biosensors, the main advantage of which is a simplified two-electrode system without an external power supply^5^. In addition to lactate^6^, cholesterol^7^, ethanol^8^ and other EBFCs, special interest in recent years has been focused on the development of membrane-less EBFCs that can deliver electrical energy using oxidation of glucose at an anode and O_2_^9,10^ or H_2_O_2_^11,12^ reduction at a cathode. Glucose and O_2_ are an ideal source of fuel and oxidizer because they are found in all organic tissues and can be constantly replenished during metabolic processes^13^. EBFCs that use enzymatic reactions on both electrodes have also been researched and published^14,15^. Researchers expect that in the future, miniature membrane-less EBFCs will supply energy to implantable medical devices, like insulin pumps, hearing devices, bone stimulators or pacemakers, and will also be used as self-powered biosensors, which, using an analyte as a fuel, will be able to supply themselves with energy, and at the same time to determine the amount of an analyte^16^. Implanted self-powered biosensors could be used to measure various substances that cause heart disease or cancer, as well as blood glucose^17,18^. To use EBFCs for these purposes, they should be small and light, operate at body temperature, pH, and salt concentration, as well as be able to generate a sufficient amount of energy and have good operational stability. Due to insufficient power output, voltage output, and operational stability, the development of high-performance EBFCs is still required.

Over the last decade, the performance of EBFCs has greatly improved by using various nanomaterials, such as carbon nanotubes (CNTs), graphene oxide (GO), noble metal nanoparticles or conjugated polymers (CPs). These materials often have good biocompatibility, also electrical conductivity and large surface area. Their use allows to improve the efficiency of electron transfer and a magnitude of the generated electrical signal and often provides a stable matrix for enzyme immobilization. Due to the large surface area, nanomaterials can increase enzyme loading, furthermore, to improve the activity and stability of immobilized enzymes, thus improving the performance of EBFCs. Among the nanomaterials mentioned, CPs and CPs-based nanocomposites have gained the considerable attention of many scientists. For example, Haque and co-workers^19^ reported a glassy carbon electrode modified with a conducting composite consisting of chitosan, reduced GO, polyaniline (PANI), ferritin and glucose oxidase (GOx) as a potential bioanode for glucose EBFC. The bioanode was capable to generate a current of 3.5 mA/cm^2^ at 20 mM of glucose. The performance was improved due to the porosity and large surface area of the composite material, which allows the immobilization of a larger amount of enzyme and facilitates the diffusion of glucose. Although the bioanode generated a lower current signal, it nevertheless had a high operational stability and maintained 95% of the initial response after one week. Kang and co-workers^20^ proposed a glucose/O_2_ EBFC based on glassy carbon electrodes modified with a novel three-dimensional PANI and CNTs composite with rhizobium-like structure. The composite was prepared by in-situ polymerization of aniline monomers around and along the functionalized CNTs and then carbonized at a high temperature was used as a substrate for immobilization of GOx (anode) and laccase (Lac) (cathode). The EBFC was performed with a maximum power density of 1.12 mW/cm^2^ at 0.45 V. Moreover, three fabricated EBFCs connected in series were able to light up a yellow light-emitting diode (LED) whose turn-on voltage was about at 1.8 V. Later Kang and co-workers^21^ reported glucose/O_2_ EBFC based on GOx and Lac immobilized on carbonized rectangular polypyrrole tubes. A nickel foam was utilized as the substrate electrode. The open-circuit voltage The open-circuit of the designed EBFC reached 1.16 V and the maximum power density was measured to 0.350 mW/cm^2^ at 0.85 V. Three of the fabricated EBFCs connected in series were able to light up a white LED whose turn-on voltage was about at 2.4 V for more than 48 h.

Most of the glucose EBFCs utilize glucose-oxidizing enzyme (GOx or glucose dehydrogenase) on a bioanode combined with oxygen reducing enzymes (commonly bilirubin oxidase or Lac) on a biocathode. Biocathodes based on immobilized peroxidase (PO)^14,22^ and biocathodes in which GOx is combined with PO that catalyses the reduction of H_2_O_2_, produced during glucose oxidation on GOx modified electrodes^23^, have also been published. Such systems have a drawback: the use of two enzymes, which makes the system more complex and increases the cost. In addition, enzymes used may have different optimal operating conditions. These drawbacks can be avoided by designing a so-called single enzyme EBFC, in which the same enzyme is used for the anodic and cathodic reactions. The present paper describes the fabrication and investigation of a novel biocathode in the construction of which an “artificial PO” Prussian blue (PB) was used instead of PO. According to the mechanism of H_2_O_2_ reduction on PB modified electrodes, PB is electrochemically reduced to form Prussian white (PW), which catalyses the reduction of H_2_O_2_ at low potential^24^ and PW is oxidized to PB again. Due to the reversible electrochemical redox ability of PB, it acts as a renewable catalyst throughout the electrochemical process^25^. Although PO-like property of PB has been studied for the design of biosensors^5,26^ and FCs^27,28^, it has not yet been used in the construction of a biocathode for a single enzyme EBFC, whose anodic and cathodic reactions would be both based on the processes biocatalysed by GOx. This enzyme immobilized on the bioanode and biocathode would catalyze the oxidation of glucose to H_2_O_2_, which would be reduced on the surface of the biocathode. To design such biocathode, graphite rod (GR) electrode was coated with a composite of PB particles embedded in the PPCA shell (GR/PB-PPCA) and an additional layer of PPCA (GR/PB-PPCA/PPCA) by cyclic voltammetry (CV). Finally, GOx was covalently linked to the carboxyl groups of the PPCA (GR/PB-PPCA/PPCA–GOx). Immobilized GOx acted as a catalyst that oxidizes glucose by molecular O_2_, PB, meanwhile, acted as an electrocatalyst for the reduction of H_2_O_2_ formed during the enzymatic reaction. To achieve the best performance of the biocathode, preparation conditions were optimized by evaluating the generated signal to glucose. After optimization, the performance of the biocathode was investigated.

## Materials and methods

### Chemicals

GOx from *Aspergillus niger* (freeze-dried powder 360 U/mg protein), iron (III) chloride (FeCl_3_) and N-(3-dimethylaminopropyl)-N′-ethylcarbodiimide hydrochloride (EDC) were purchased from Carl Roth GmbH. Potassium hexacyanoferrate (III) (K_3_[Fe(CN)_6_]) were from Sigma-Aldrich. Pyrrole-2-carboxylic acid (PCA) and D-(+)-glucose monohydrate (C_6_H_12_O_6_ × H_2_O) were obtained from Alfa Aesar GmbH. Hydrogen peroxide (H_2_O_2_) was obtained from Chempur and N-hydroxysuccinimide (NHS) from Merck. All chemicals were of analytical grade. All aqueous solutions were prepared in ultra-high quality (UHQ) water, which was obtained using the DEMIWA rosa 5 water purification system (WATEK, Czech Republic). In addition, glucose solution was prepared at least 24 h before use to allow glucose to mutarotate and to reach equilibrium between α- and β-forms. 40.0 mg/mL solution of GOx was freshly prepared in sodium acetate-phosphate buffer solution composed of 0.05 mM CH_3_COONa, 0.05 mM Na_2_HPO_4_ and 0.05 mM NaH_2_PO_4_ (A-PBS) and rapidly used. 0.5 M solution of PCA was prepared in ethanol absolute.

### Instrumentation

All electrochemical experiments as well as electrochemical synthesis of the PB-PPCA/PPCA composite were performed using a computer-controlled potentiostat/galvanostat Autolab PGSTAT30 (Eco Chemie, Netherlands) driven by NOVA1.9 software. The voltammetric and amperometric cell was composed of Pt wire electrode as a counter electrode, Ag/AgCl (3.0 M of KCl) reference electrode, and GR or modified GR electrode as a working electrode. The potentiometric cell was composed of Ag/AgCl electrode and a modified GR electrode. All experiments were carried out inside a Faraday-cage at ambient temperature. Morphological studies of GR and modified GR electrodes were performed using scanning electron microscope (SEM) Helios Nanolab 650 (FEI, Netherlands).

### GR electrode pre-treatment and preparation of the biochatode

GR electrodes were prepared by breaking a graphite rod (15.0 cm in length, 3.0 mm in diameter and 99.999% purity, Sigma-Aldrich) into smaller rods of the required length. The broken rods were mechanically polished using very fine (P320) and finally ultrafine grit (P2000) sandpaper until the working surface of the electrode is completely smooth, and then were polished using a sheet of paper, washed with UHQ water, then ethanol and dried at room temperature. Finally, the side surface of the rod was isolated with a silicone tube so that only the working surface of the GR electrode was in contact with the solution in the electrochemical cell. The working surface area of GR electrodes thus prepared was 0.0707 cm^2^.

Pre-treated GR electrodes were used to perform electrochemical synthesis of the PB-PPCA/PPCA composite using CV. Under the optimized modification conditions, the pre-treated GR electrode, together with the reference and auxiliary electrodes, was immersed in an electrochemical cell filled with 5 mL of a solution consisting of 100.0 mM HCl, 100.0 mM KCl, 1.0 mM FeCl_3_, 1.0 mM K_3_[Fe(CN)_6_] and 35.0 mM PCA. The potential was then scanned for 50 consecutive cycles in the range of potentials from − 0.4 to + 1.0 V at a scan rate of 0.1 V/s. During this process, a composite of PB particles embedded in the PPCA shell (PB-PPCA) was formed on the GR electrode surface (GR/PB-PPCA). After synthesis of the PB-PPCA composite, the GR/PB-PPCA electrode was washed well with UHQ water and immersed in an electrochemical cell filled with 5 mL of A-PBS buffer solution with 0.1 M KCl additive (A-PBS-KCl), pH 6.0, and containing 200.0 mM PCA. The potential was then scanned for 5 consecutive cycles in the range of potentials from − 0.4 to + 1.0 V at a scan rate of 0.1 V/s. During this process, an additional layer of PPCA was formed on the GR/PB-PPCA electrode (GR/PB-PPCA/PPCA).

To modify the GR/PB-PPCA/PPCA electrode with GOx, the electrode was immersed in a tube filled with a mixture of 0.4 M EDC and 0.1 M NHS solutions in a ratio of 1:1 and left for 30 min at ambient temperature. The electrode was then removed from the mixture, washed with UHQ water, and immersed immediately in a 40 mg/mL GOx solution in A-PBS, pH 4.0, stirring the solution gently from time to time. The GOx was attached covalently by an amide bond to the electrode surface (GR/PB-PPCA/PPCA–GOx). Finally, the GR/PB-PPCA/PPCA–GOx electrode was washed with UHQ water and to remove non-covalently bound enzyme was conditioned in A-PBS-KCl, pH 6.0, for 15 min, stirring the solution gently from time to time. The prepared GR/PB-PPCA/PPCA–GOx electrodes were stored in closed test tubes above a drop of A-PBS-KCl, pH 6.0, at + 4 °C temperature until used in the experiments.

### Electrochemical measurements

Electrochemical characterization of bare GR and modified GR electrodes was performed by CV, amperometric and potentiometric techniques. For half-cell measurements, three-electrode cell was used for CV and amperometric measurements, while two-electrode cell was used for potentiometric measurements at open circuit or at an external load of 476 kΩ. A-PBS-KCl buffer solution, pH 6.0, was used as the electrolyte solution. The solution in the cell was continuously stirred with a magnetic stirrer at 450 rpm during amperometric and potentiometric measurements. Meanwhile, CV measurements were performed without stirring; stirring was turned on only after the addition of glucose to mix the solution. Amperometric current dependence of the biocathode on glucose concentration was studied at + 100 mV vs reference electrode. After stabilization of the background current or potential (base line), in the amperometric and potentiometric measurements respectively, a solution of glucose was injected in the electrochemical cell. The biocathode-generated signal was expressed as the change in cathodic current (ΔI) or the change in potential (ΔE) calculated from the signal recorded by the addition of glucose minus the baseline signal. During the CV measurements, the potential was scanned in the range of potentials from − 0.2 to + 0.5 V at a scan rate of 0.1 V/s or other as required, and the peak current was monitored. The results of all experiments were represented as a mean value of three independent measurements.

## Results and discussion

**Figure 1:**
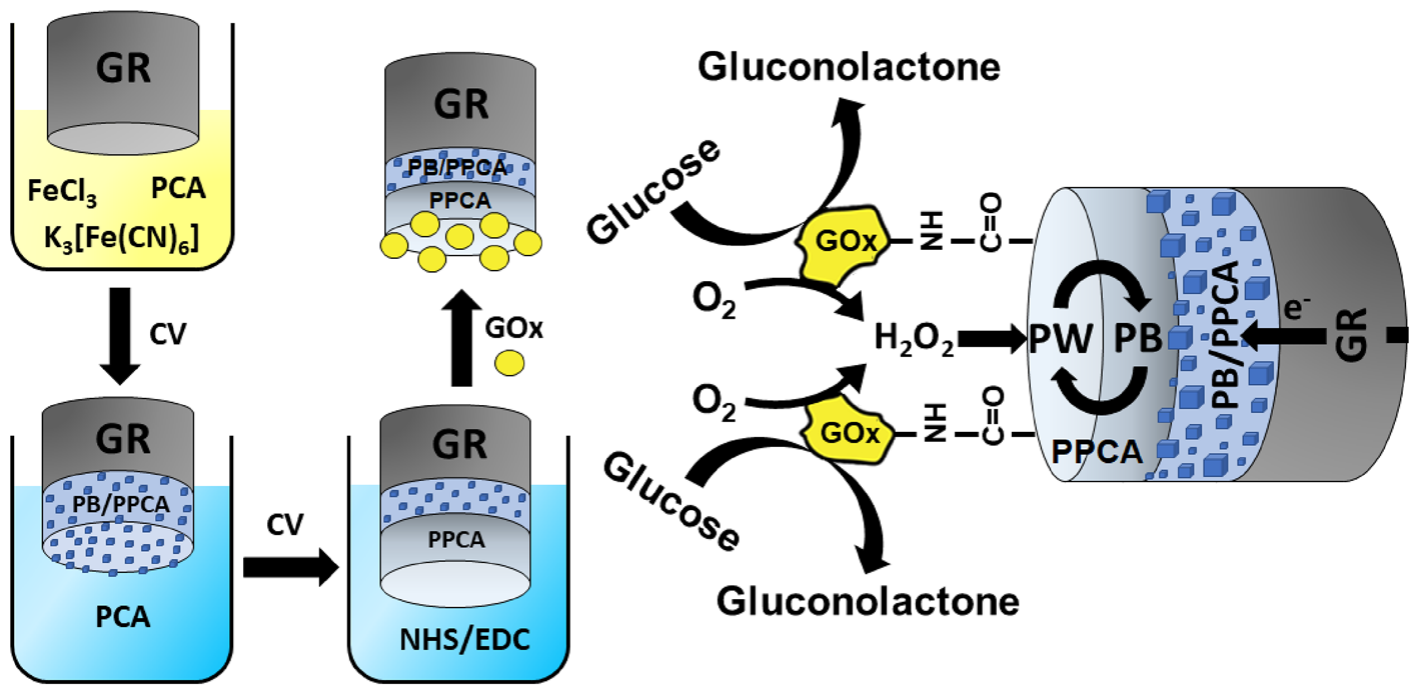
Scheme demonstrating fabrication and operation of the GR/PB-PPCA/PPCA–GOx biocathode.

In this research, a novel GR/PB-PPCA/PPCA–GOx biocomposite based biocathode was developed. CV was used for the electrochemical synthesis of the composite consisting of PB particles embedded in a PPCA shell (PB-PPCA) and for the formation of an additional PPCA layer over PB-PPCA (PB-PPCA/PPCA). To synthesize PB-PPCA on top of a GR electrode (GR/PB-PPCA), the electrode was immersed in an electrochemical cell filled with a solution consisting of HCl, KCl, FeCl_3_, K_3_[Fe(CN)_6_] and PCA, and polymerization was performed. The GR/PB-PPCA electrode was then immersed in an electrochemical cell filled with A-PBS-KCl buffer solution containing PCA and an additional PPCA layer (GR/PB-PPCA/PPCA) was synthesized. Finally, using activation of carboxyl groups of PPCA by a mixture of EDC and NHS, GOx molecules were linked to the PPCA via amide bonds (GR/PB-PPCA/PPCA–GOx). The design concept and operation of the biocathode are shown in Fig. 1. The operation of the biocathode can be explained by the electrocatalytic activity of PB towards to the reduction of H_2_O_2_ formed during GOx catalyzed oxidation of glucose. Fe(III) of PB after receiving the electron is electrochemically reduced to form PW, which has a high reduction activity^25^. The H_2_O_2_ formed during enzymatic reaction is reduced by PW, and the PW is reoxidized to PB. Due to the reversible redox activity of PB, it acts as a renewable catalyst throughout the bioelectrochemical process. The amount of H_2_O_2_ formed during GOx-catalyzed reaction depends on the glucose concentration, thus the current or potential signal generated by the biocathode due to H_2_O_2_ reduction dependent on the glucose concentration.

### Optimization of the biocathode preparation conditions

**Figure 2:**
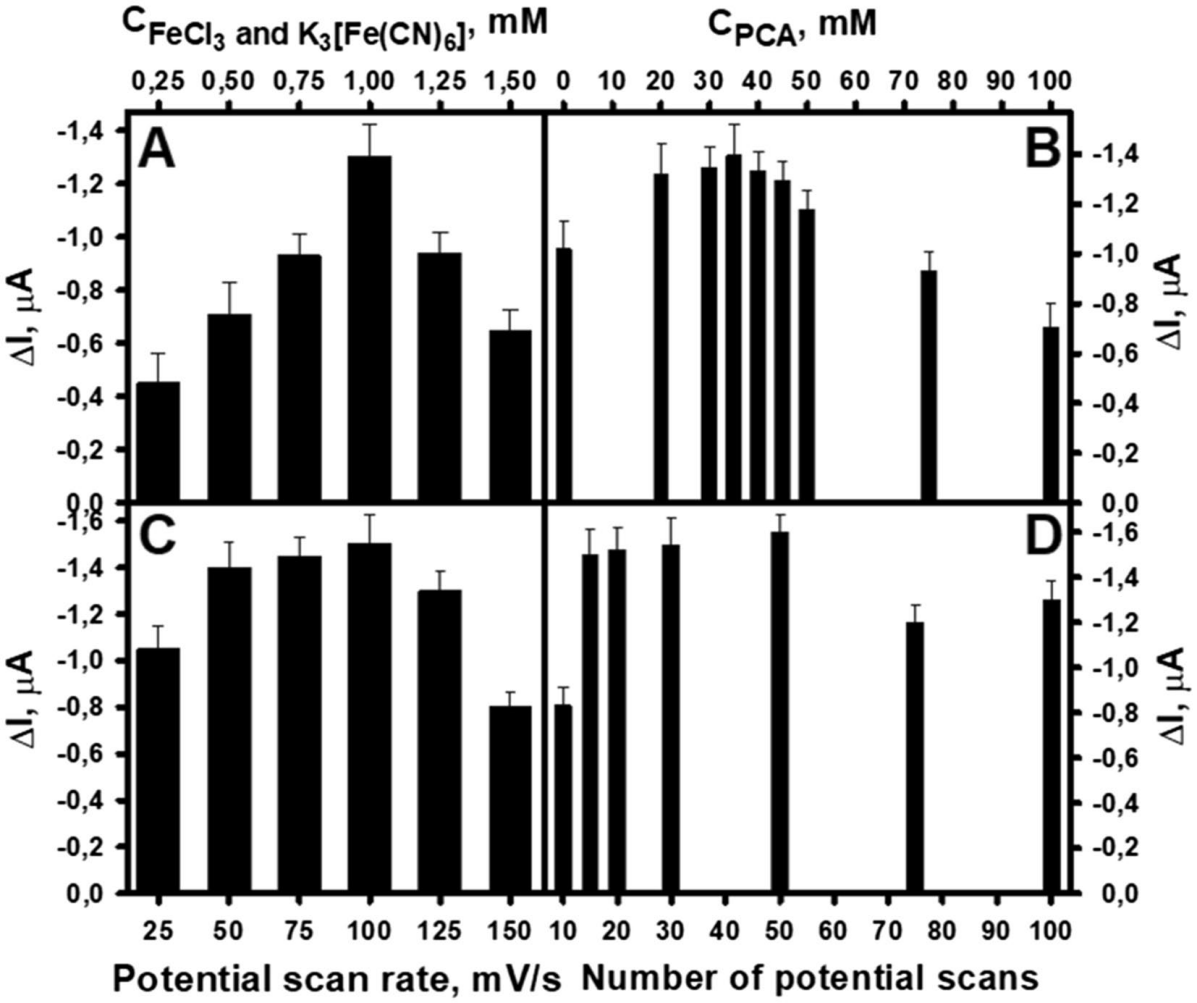
Optimization of electrochemical PB-PPCA synthesis conditions: concentration of FeCl_3_, K_3_[Fe(CN)_6_] **(A)** and PCA **(B)**, potential scan rate **(C)** and number of scans **(D)**. Constant experimental conditions: 45.0 mM of PCA, 15 scans at potential scan rate of 0.05 V/s (A); 1.0 mM of FeCl_3_ and K_3_[Fe(CN)_6_], 15 scans at potential scan rate of 0.05 V/s (B); 1.0 mM of FeCl_3_ and K_3_[Fe(CN)_6_], 35.0 mM of PCA, 15 scans (C); 1.0 mM of FeCl_3_ and K_3_[Fe(CN)_6_], 35.0 mM of PCA, potential scan rate of 0.10 V/s (D). Experimental conditions for the synthesis of an additional PPCA layer: 100.0 mM of PCA, potential scan rate of 0.1 V/s, 10 scans. GOx concentration: 40.0 mg/mL at pH 4.0. Measurement conditions: applied potential of + 0.1 mV, A-PBS-KCl, pH 6.0, 7.97 mM of glucose.

To obtain the best performance of the biocathode, its preparation conditions were optimized by estimating the magnitude of the generated current signal to glucose. Since the GR electrode was coated layer by layer with PB-PPCA/PPCA composite, the electrochemical synthesis conditions of PB-PPCA were first optimized. During optimization, PB-PPCA electrodes prepared under different conditions were modified with an additional layer of PPCA and immobilized GOx under constant conditions. The amperometric response of the GR/PB-PPCA/PPCA–GOx biocathodes to glucose in A-PBS-KCl buffer solution, expressed as cathodic current change (ΔI), was then investigated. Figure 2A shows the experimental results obtained during the optimization of FeCl_3_ and K_3_[Fe(CN)_6_] concentration. As can be seen, the current signal increased with increasing equimolar concentrations of FeCl_3_ and K_3_[Fe(CN)_6_] up to an optimal concentration of 1.0 mM. Meanwhile, the optimal PCA concentration was found to be 35.0 mM (Fig. 2B). It is likely that when the concentrations of FeCl_3_ and K_3_[Fe(CN)_6_] are too high and the PCA is too low, PB particles from the resulting PB-PPCA composite can diffuse into the solution, thus reducing the current generated by the biocathode. On the other hand, at too high concentration of PCA, a thick layer of PPCA is formed. Due to the low conductivity of this layer, the current generated is also reduced.

The current response to glucose was also depended on the potential scan rate (Fig. 2C) and the number of potential scans (Fig. 2D). The highest current response of the biocathode after addition of glucose was registered when a potential scan rate of 0.1 V/s and 50 potential scans were used. During polymerization, a thick polymer shell is formed by applying more potential scans and higher scan rate. Meanwhile, with less potential scans and lower scan rate, the electrode surface may be inefficiently coated by PB-PPCA composite. This, as can be seen from the results, also affects the current generated by the biocathode.

**Figure 3:**
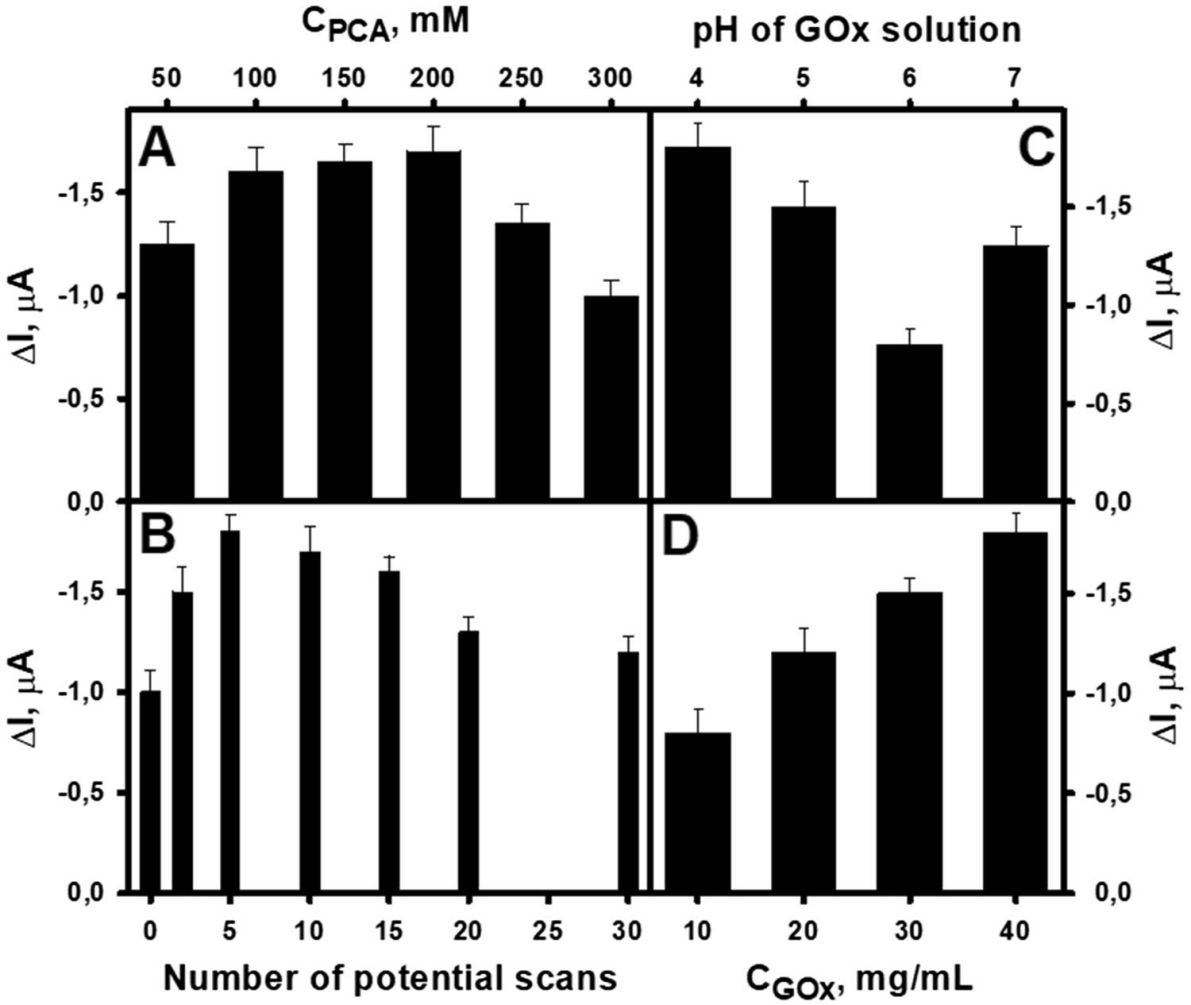
Optimization of additional PPCA layer electrochemical synthesis and GOx immobilization conditions. **(A)** Optimization of PCA concentration. Constant experimental conditions: potential scan rate of 0.1 V/s, 10 scans. **(B)** Optimization of the number of scans. Constant experimental conditions: 200.0 mM of PCA, potential scan rate of 0.1 V/s. Optimization of GOx solution pH **(C)** and concentration **(D)**. Experimental conditions for the synthesis of PB-PPCA: 1.0 mM of FeCl_3_ and K_3_[Fe(CN)_6_], 35.0 mM of PCA, potential scan rate of 0.1 V/s, 50 scans. GOx concentration: 40.0 mg/mL at pH 4.0 (A – C). GOx solution pH: 4.0 (A, B, D). Measurement conditions: applied potential of + 0.1 V, A-PBS-KCl, pH 6.0, 7.97 mM of glucose.

During the optimization of the deposition of the additional PPCA layer, the dependence of the current signal on PCA concentration and potential scan rate was also observed. The highest current signal was registered when 200 mM of PCA (Fig. 3A) and 5 potential scans (Fig. 3B) were used. Such results are related to the thickness of the resulting additional PPCA layer. The higher the PCA concentration and the number of potential scans, the thicker the additional PPCA layer. The thicker layer causes a decrease in the current generated by the biocathode. On the other hand, with a fuller and more uniform coating, more GOx molecules can be attached to the electrode surface. Based on the results of this study, 200 mM PCA and 5 potential scans were selected as optimal conditions for the electrochemical polymerization of the additional PPCA layer on the GR/PB-PPCA electrode.

The concentration and pH of the GOx solution used for biocathode preparation were also optimized. In this work, a covalent amide coupling technique using a mixture of EDC and NHS was used for GOx immobilization. To find the most suitable pH, after activation of the PPCA carboxyl groups, the GR/PB-PPCA/PPCA electrodes were immersed in GOx solution in A-PBS buffer with a certain pH from 4.0 to 7.0. The current response of the prepared biocathodes to glucose was then investigated. As can be seen from the results presented in Fig. 3C, the magnitude of the current signal was dependent on the pH, and the highest current signal was registered at pH 4.0. These results were consistent with those obtained for GR electrodes modified with a nanobiocomposite composed of poly(1,10-phenanthroline-5,6-dione), PPCA, gold nanoparticles and GOx^29^, and can be explained by the pre-concentration of the enzyme near the electrode surface at this pH, resulting in a higher amount of immobilized GOx. Very similar results were obtained when the influence of the concentration of GOx solution used during immobilization on the magnitude of the current signal generated by the biocathode was investigated. The increase in the enzyme concentration resulted in an increase in the current response of the biocathode to glucose and the highest response was recorded then the highest GOx concentration of 40 mg/mL was used (Fig. 3D). Because 40 mg/mL is a sufficiently high concentration, the effect of higher concentrations was not studied and this GOx concentration was chosen for use in biocathode preparation.

### Electrochemical behaviour of the biocathode

**Figure 4:**
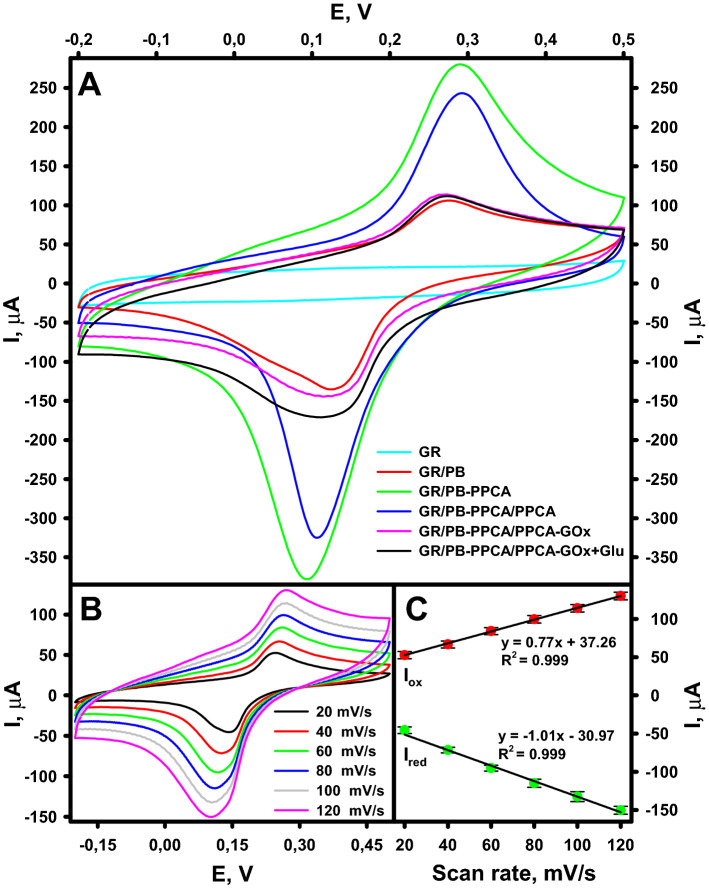
**(A)** Cyclic voltammograms for electrodes obtained after an appropriate step of the biocathode preparation process recorded in pure A-PBS-KCl, pH 6.0, or in 10 mM glucose (Glu) solution in A-PBS-KCl, pH 6.0, at a scan rate of 100 mV/s. **(B)** Cyclic voltammograms for biocathode recorded in A-PBS-KCl, pH 6.0, at various scan rates. **(C)** Plots of peak current vs scan rate.

Electrochemical behaviour was studied by recording cyclic voltammograms after an appropriate step of the biocathode preparation process. Figure 4A shows the corresponding cyclic voltammograms registered for GR, GR/PB, GR/PB-PPCA, GR/PB-PPCA/PPCA, and GR/PB-PPCA/PPCA–GOx electrodes. As can be seen, in the voltammogram recorded for the GR electrode, there are no oxidation and reduction current peaks (redox peaks) in a potential range between − 0.2 and + 0.5 V vs Ag/AgCl. Meanwhile, for the other electrodes studied, a pair of well-defined redox peaks due to the electrochemical reaction of high-spin ferric ions in PB (Fe^2+^/Fe^3+^ transition)^24^ were recorded in this potential range. The positions of the characteristic redox peaks and the distances between them are given in Table 1.

**Table 1:** Voltammetric parameters of different modified GR electrodes obtained in A-PBS-KCl, pH 6.0, at a scan rate of 0.1 V/s.

Electrode	E_ox_, mV	E_red_, mV	ΔE, mV	I_ox_, μA	I_red_, μA
GR/PB	277.8 ± 4.1	120.7 ± 4.4	155.6 ± 3.8	109.1 ± 7.1	– 128.4 ± 9.5
GR/PB-PPCA	290.6 ± 4.9	93.3 ± 5.9	197.2 ± 1.1	268.5 ± 12.7	– 362.1 ± 14.2
GR/PB-PPCA/PPCA	291.3 ± 5.3	104.0 ± 4.1	187.3 ± 1.3	232.8 ± 10.9	– 311.7 ± 17.1
GR/PB-PPCA/PPCA–GOx	272.4 ± 6.1	103.5 ± 4.6	168.9 ± 3.9	114.1 ± 12.4	– 135.6 ± 15.0

The occurrence of characteristic redox peaks and the increase in peak current compared to the GR electrode indicate successive deposition of PB on all modified electrodes. The observed redox peaks potential values were similar to those described in the literature for PB modified electrodes^30–32^. In addition, as can be seen from Fig. 4A, the cyclic voltammograms of the GR/PB-PPCA, GR/PB-PPCA/PPCA and GR/PB-PPCA/PPCA–GOx electrodes showed a significant increase in redox peaks intensity compared to the GR/PB electrode. These results demonstrated that the presence of PCA in the electropolymerization solution increases the amount of PB on the electrode surface due to its distribution inside the polymer matrix. Meanwhile, the decrease in the intensity of the GR/PB-PPCA/PPCA redox peaks compared to GR/PB-PPCA could be explained by the formation of an additional PPCA layer. Because electropolymerization was carried out in an aqueous medium without removal of oxygen, the PPCA layer formed is low conductive or even non-conductive^29^. Therefore, the formation of an additional layer of PPCA causes a decrease in the intensity of the redox peaks. For the GR/PB-PPCA/PPCA–GOx, an even greater decrease in redox peaks intensity was observed due to nonconducting nature of enzyme^33,34^ immobilized on the surface. In addition, as shown in Fig. 4A, the presence of 10.0 mM glucose in A-PBS-KCl buffer solution caused an increase in GR/PB-PPCA/PPCA–GOx reduction peak current by approximately 29 μA (black line). This indicates that the H_2_O_2_ formed during the enzymatic glucose oxidation reaction was electrochemically reduced on the surface of the biocathode.

The cyclic voltammograms of the GR/PB-PPCA/PPCA–GOx biocathode in A-PBS-KCl, pH 6.0, at different potential scan rates are shown in Fig. 4B. As can be seen, the intensity of the redox peaks varied with the potential scan rate and was directly proportional to the scan rate (Fig. 4C). The linear relationship between oxidation (I_ox_) and reduction (I_red_) current peaks and potential scan rate and the ratio of I_ox_/I_red_ almost equal to unity, revealed the quasi-reversible and surface-confined^32^ electrochemical behaviour of the PB in PB-PPCA/PPCA–GOx biocomposite, in which PB is reduced to PW and is re-oxidized to PB.

### Morphological study

**Figure 5:**
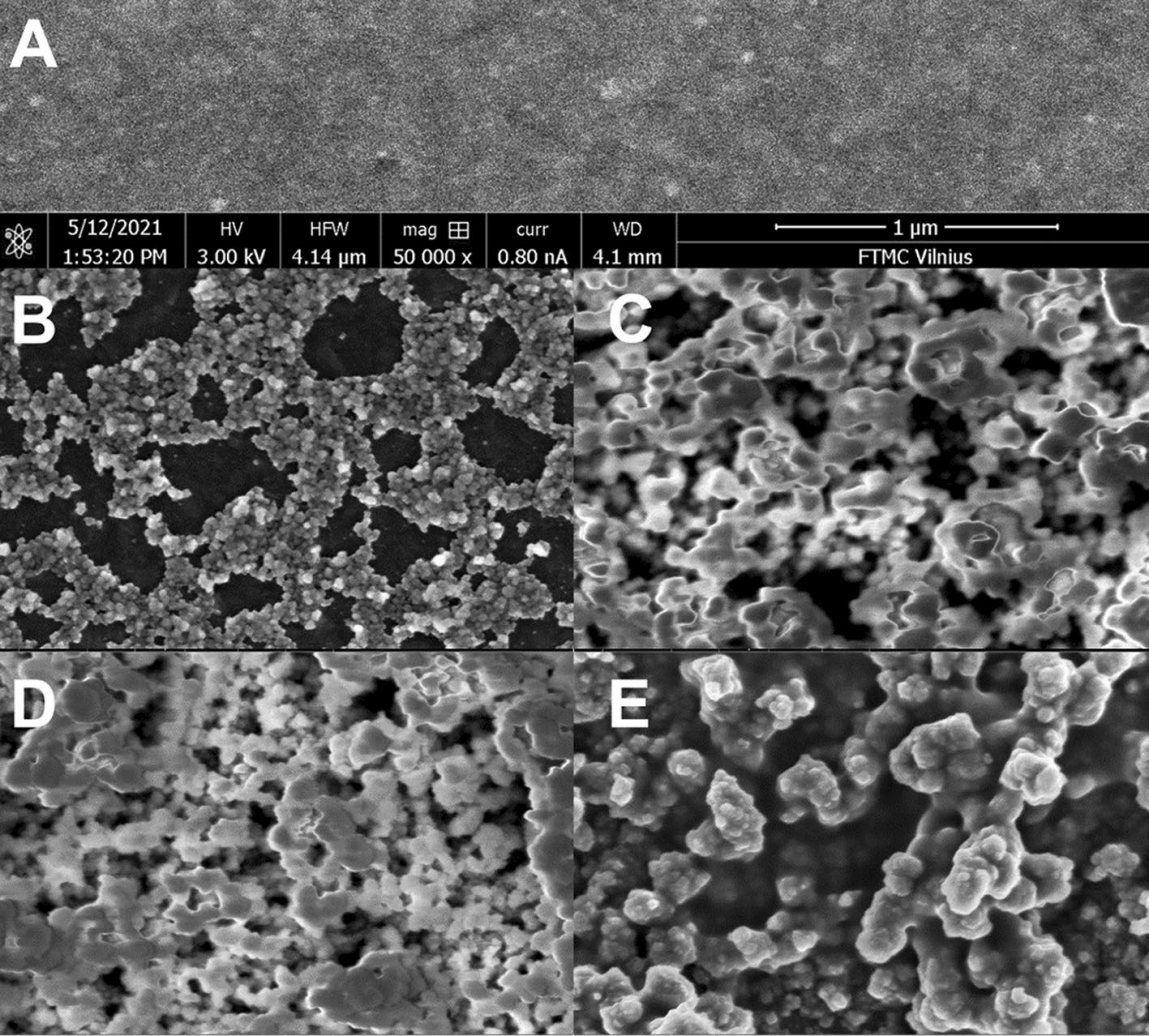
SEM images of GR (**A**) and GR/PB (**B**), GR/PB-PPCA (**C**), GR/PB-PPCA/PPCA (**D**) and GR/PB-PPCA/PPCA–GOx (**E**) modified electrodes.

The surface morphology of bare GR and GR at different stages of the modifying process (GR/PB, GR/PB-PPCA, GR/PB-PPCA/PPCA, and GR/PB-PPCA/PPCA–GOx) was studied using SEM at 3 kV accelerating voltage, 50,000 magnification and 0.8 nA current. The SEM images in Fig. 5 clearly demonstrate changes in surface morphology during GR modification.

As can be seen, the GR surface is quite smooth with minor defects occurring during surface polishing. (Fig. 5A). Meanwhile, a completely different surface morphology was observed for the other electrodes. Cubic PB structures of approximately 100 nm in size are observed on the surface of GR/PB (Fig. 5B), similar to those published by other authors^32,35^. However, as can be seen, the surface coating is very uneven with large, uncoated GE areas. Not uniform coating may be related to the removal of PB particles from the surface during synthesis and electrode washing after synthesis. Meanwhile, the presence of PPCA in the synthesis solution resulted in a much better and more uniform coating with a higher amount of PB on the electrode surface (Fig. 5C). However, the characteristic cubic structure of PB was no longer visible. This change in morphology may be related to the disruption of the growth of PB particles into the cubic framework due to the spatial limitations resulting from the embedment of PB particles into the growing PPCA shell^36^. PB-PPCA coating showed an irregular globular morphology, which was in agreement with other reports for PB and polymer composites^36,37^. A rougher coating with larger structures compared to GR/PB-PPCA as well as globular surface morphology was observed for GR/PB-PPCA/PPCA (Fig. 5D). This indicates that PB-PPCA was coated with an additional layer of PPCA. Similar surface morphology, with slightly larger structures was observed for GR/PB-PPCA/PPCA–GOx (Fig. 5E). The results of surface morphology studies confirmed the assumption that PB was incorporated into PPCA during the formation of the PB-PPCA layer, and an additional PPCA layer was formed on the surface of the PB-PPCA.

### Study of the biocathode response to glucose

**Figure 6:**
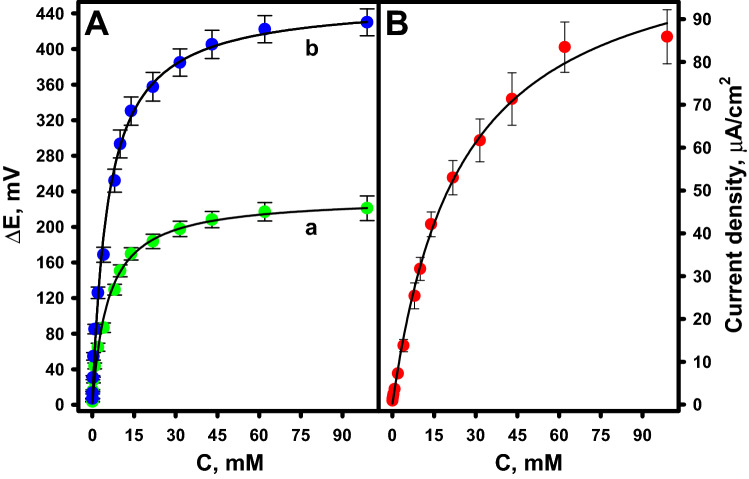
Dependence of potential (**A**) and current density (**B**) at open-circuit (curve b) and at an external load of 476 kΩ (curve a) generated by the biocathode on glucose concentration. Measurements conditions: A-PBS-KCl, pH 6.0, applied potential of + 0.1 V for amperometric measurements.

Figure 6 shows the influence of glucose concentrations on the reduction current, the potential at an external load of 476 kΩ (curve a) and the open-circuit potential (OCP) (curve b) generated by the GR/PB-PPCA/PPCA–GOx biocathode.

Three-electrode cell consisting of Pt wire counter electrode, Ag/AgCl reference electrode, and GR/PB-PPCA/PPCA–GOx biocathode was used for current measurements, while two-electrode cell consisting of Ag/AgCl reference electrode and GR/PB-PPCA/PPCA–GOx biocathode was used for potentiometric measurements at open circuit or at an external load of 476 kΩ. The measurements were performed in O_2_-saturated A-PBS-KCl solution, pH 6.0, at different glucose concentrations from 0.05 to 98.86 mM. The error bars correspond to the values of each concentration recorded by the three different biocathodes and illustrate the reproducibility of the measurements and the fabrication of the biocathode.

As can be seen, a linear increase in biocathode response was observed with increasing glucose concentration up to about 10.00 mM. With further increase in glucose concentration, a non-linear increase in the biocathode response was observed up to 98.86 mM, and then the recorded signal became saturated because the catalytic reaction was inhibited at higher glucose concentrations. As a result, the amount of H_2_O_2_ formed during the enzymatic reaction became constant. The maximal current density of 85.86 ± 6.30 μA/cm^2^, the potential of 221.03 ± 13.90 mV, and the OCP of 430.15 ± 15.10 mV at 98.86 mM glucose were recorded. Meanwhile, at 10.00 mM glucose, the recorded current density was 31.68 ± 2.70 μA/cm^2^, the potential was 150.73 ± 6.70 mV and the OCP was 293.34 ± 15.70 mV. The performance of the developed biocathode was comparable to the previously reported biocathodes. Some of them are listed in Table 2.

**Table 2:** Comparison of the performance of some biocathodes described in the literature with the biocathode developed in this work.

Biocathode	OCP, mV	Current density, μA/cm^2^	References
MWBP/BOx	520 vs Ag/AgCl	–	^38^
CNBP/Lac	557 vs SCE	50.0	^39^
PGE/PANI/MWCNT/Lac	528 vs Ag/AgCl	1209.2	^40^
MWCNT/Lac	430 vs SCE	–	^41^
DWCNT/HRP/GOx	380 vs SCE	115	^23^
GR/MWCNT/HRP/GOx	550 vs Ag/AgCl	–	^42^
SPE/PB/ChOx	–	8.8	^5^
CNBP/PTH/BOx	520 vs Ag/AgCl	–	^43^
AlO/CoNT/PANI/Lac	590 vs Ag/AgCl	140.12	^44^
P2/BOx/PEGDGE	420 vs Ag/AgCl	–	^45^
GR/PB-PPCA/PPCA–GOx	430 vs Ag/AgCl	85.86	This work

MWBP - multi-walled carbon nanotube paper; BOx - bilirubin oxidase; CNBP - carbon nanotube buckypaper; PGE - pencil graphite electrode; MWCNT - multi-walled carbon nano tubes; MWCNT - double-walled carbon nano tubes; HRP - horseradish peroxidase; SPE - screen-printed electrode; ChOx - cholesterol oxidase; PTH - polythiophene; AlO - anodic aluminium oxide template; CoNT - copper nanotubes; PEGDGE - poly(ethylene glycol) diglycidyl ether; P2 - poly(1-vinylimidazole-co-[2-(methacryloyloxy)ethyl]trimethylammoniumchloride)-[Os(bpy)_2_Cl]^+^

.

### Stability study of the biocathode

**Figure 7:**
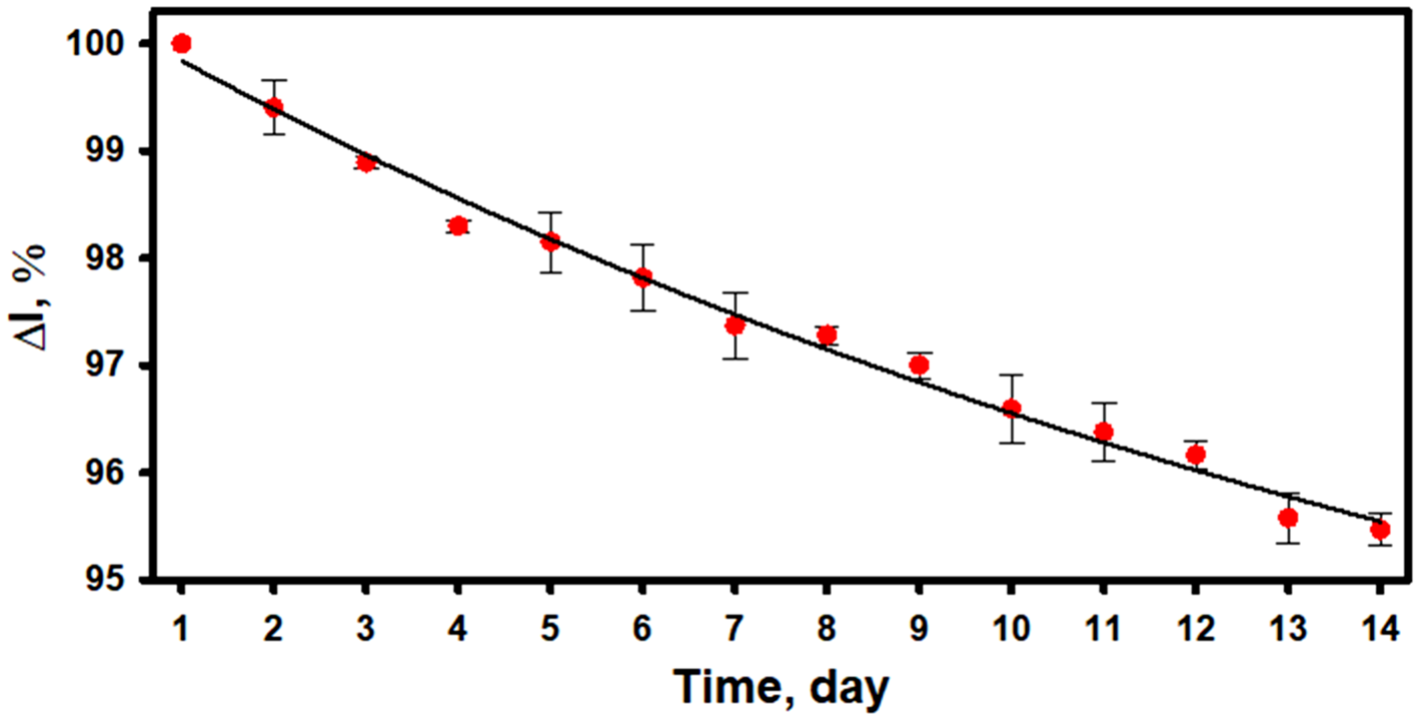
Changes of the biocathode current signal vs time. Measurements conditions: A-PBS-KCl, pH 6.0, 10.0 mM of glucose, applied potential of + 0.1 V.

To investigate the stability of the developed biocathode, the current signal generated by it was monitored over a period of 14 days. The measurements were carried out in A-PBS-KCl buffer solution, pH 6.0, containing 10.0 mM of glucose. Between measurements, the biocathode was stored at + 4 °C in a closed vessel above a drop of A-PBS-KCl buffer solution, pH 6.0. As can be seen from the experimental data presented in Fig. 7, current generated by the GR/PB-PPCA/PPCA–GOx biocathode after addition of glucose gradually decreased over a period of 14 days. However, the biocathode maintained 95.53 ± 0.15% of the initial response after 14 days. Such a relatively small decrease in current response may be associated with the covalent immobilization of GOx^46^ and the stability of PB-PPCA/PPCA composite.

## Conclusion

In this study, a graphite rod electrode modified with a biocomposite composed of Prussian blue, poly(pyrrole-2-carboxylic acid) and glucose oxidase (GOx) was proposed as a potential biocathode for glucose-powered single enzyme biofuel cell. PPCA allowed covalent immobilization of GOx, PB, meanwhile, exhibited electrocatalytic activity to the reduction of H_2_O_2_ formed during GOx-catalysed oxidation of glucose. The developed biocathode proved to be able to generate a current density of 31.68 ± 2.70 μA/cm^2^ and open-circuit potential of 276.74 ± 15.70 mV in O_2_-saturated 10 mM glucose-buffered solution at pH 6.0. A maximum open-circuit potential of 405.80 ± 15.10 mV was recorded at 98.86 mM glucose. In addition, the biocathode showed good operational stability, maintaining 95.53 ± 0.15% of the initial current response after 14 days. This study demonstrates the potential of the designed biocathode to develop glucose-powered single enzyme biofuel cell, therefore, further work will focus on its development. The advantage of such biofuel cells is that they do not require two enzymes, which simplifies the system, reduces costs and lead to avoid disparities in the operating conditions of the enzymes used. It is likely that the use of the designed biocathode will improve the performance of the biofuel cell to be developed. In addition, this simple biocathode design can serve to develop single enzyme biofuel cells based on other oxidases.
